# Clinical effect of locking compression plate via posterolateral approach in the treatment of distal femoral fractures: a new approach

**DOI:** 10.1186/s13018-018-0756-7

**Published:** 2018-03-16

**Authors:** Wenzhao Xing, Wei Lin, Jia Dai, Zhigang Kong, Yanfeng Wang, Lei Sun, Zhiguo Zhang, Liang Sun

**Affiliations:** 1 Department of Orthopaedics, The Third Hospital of Hebei Medical University, No. 139 Ziqiang Road, Shijiazhuang, 050051 China; 2 Division of Medical Service, Hebei General Hospital, No. 348 Heping West Road, Shijiazhuang, Hebei 050051 China; 3 Department of Orthopaedics, Cangzhou People’s Hospital, No. 7 Qingchi Avenue, Cangzhou, Hebei 061000 China

**Keywords:** Femoral fracture, Internal fixation, Posterolateral approach, Locking compression plate

## Abstract

**Background:**

Distal femur fractures are difficult to manage, and the selection of implant approach for internal fixation remains controversial. This study explores the clinical outcome of treating distal femoral fractures with a locking compression plate using a posteriolateral novel approach.

**Methods:**

Twenty patients with distal femoral fractures were included in our study, and all patients underwent fixation of the fracture using a locking compression plate through a posterolateral approach. The postoperative fracture healing time, complications, and functional recovery were observed and recorded. The joint function was categorized according to the Kolmert functional criteria.

**Results:**

All patients were followed up for an average of 12 months, and all incisions healed by first intention. Among the all patients, 19 patients achieved fracture healing 3 to 4 months after surgery. The remaining 1 patient with distal femoral C_3_ comminuted fracture achieved partial fracture healing 15 months after surgery, and bone grafting was needed. All knees can reach the state of straightening, and the postoperative excellent rate was 90%. Among them, 8 patients had maximal flexion of more than 120°, 10 patients had flexion between 90° and 120°, and 2 other patients had flexion of 70° and 40°.

**Conclusions:**

Fixation of the fracture using a locking compression plate through a posterolateral approach seemed to be an acceptable surgical option for treatment of distal femoral fractures.

## Background

Fractures of the distal femur are severe injuries that present many clinical challenges to the orthopedic surgeon [1, 2]. These fractures are often unstable and comminuted and tend to occur in elderly or multiply injured patients [2]. For long, gold standard treatment modality for fixation of the distal femur fractures was angle blade plate (ABP), compression screw, and side plate devices such as dynamic condylar screw (DCS). Insertion of blade plates is technically demanding; DCS and ABP require removal of a large amount of bone for insertion; condylar buttress plates (CBP) lack the stability of fixed angle devices and are prone to varus collapse or screw failure [3, 4]. Retrograde intramedullary nails (IMNs) were not sufficient for stabilizing fragmented articular fractures [5, 6]. Nowadays, anatomically contoured locking plates and locking screws are being used more commonly for surgical fixation during distal femur fractures.

Current generation of distal femoral locking compression plates is pre-contoured based on the average bony anatomy of the adult population and they form a fixed angled construct. The pull-out strength of locking screws is higher than the conventional screws and is particularly useful in osteoporotic bones. These plates are designed to apply in minimally invasive fashion to preserve local biology and avoid fracture healing and infection problems [7, 8].

Various treatment methods have been used for distal femoral fractures. They include direct-indirect reduction, open-minimal invasive approaches, and open-slipped techniques. Condylar plates, dynamic condylar screws, condylar buttress plates, anterograde nails, retrograde nails, internal fixators, and external fixators play a more and more important role in the treatment of distal femur fractures [9]. Good fixation outcomes depend on bone quality, fracture complexity, and surgical techniques. Reddy and Chary [10] used anterolateral parapatellar approach or lateral approach for the treatment of distal femoral fractures. Anteriormiddle approach [11] and a new anterolateral approach [12] of distal femur for treatment of distal femoral fractures were reported previously. However, no reports have been reported on the treatment of distal femoral fractures by a locking compression plate via a posterolateral approach so far.

Based on the study above, our study was designed to explore the superiority of the posterior lateral approach for a locking compression plate in the treatment of distal femoral fractures.

## Methods

### Patients

We retrospectively collected the clinical data of patients with distal femoral fractures who underwent application of a locking compression plate through a posterolateral approach from March 2010 to May 2014. Ethical approval was given by the Medical Ethics Committee of the Third Hospital of Hebei Medical University. Written informed consent was obtained from all study participants.

Inclusion criteria were as follows: (1) type A and type C distal femoral fractures and (2) patients older than 18 years of age.

Exclusion criteria include as follows: (1) type B distal femoral fractures; (2) patients with poor physical conditions; (3) patients contraindicated for surgery or anesthesia; (4) patients who had wound infection prior to internal fixation; (5) patients with chronic osteomyelitis or malignant tumors; and (6) patients with concomitant neurovascular injury.

### Preoperative preparation

**Fig. 1:**
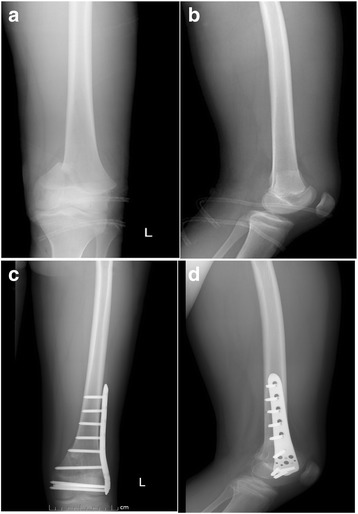
Preoperative and immediate postoperative images of X-rays. **a**, **b** Preoperative X-rays anteroposterior and lateral views of knee joint showing distal femur fracture. **c**, **d** Immediate postoperative anteroposterior and lateral X-rays showing fracture consolidation

Radiological evaluation included anteroposterior and lateral X-rays of the femur with knee, along with a pelvic X-ray to rule out proximal fractures. Computed tomography (CT) scans (Fig. 1a, b) were done to better delineate the fracture anatomy and to allow detailed subgroup classification. Chest X-ray, ECG, and laboratory tests were performed to evaluate the patients’ surgical tolerance. Patients with contraindications to surgery were excluded. All fractures were classified according to AO/OTA, and an operative proposal was made on the basis of pre-operation examination results.

### Surgical procedure

**Fig. 2:**
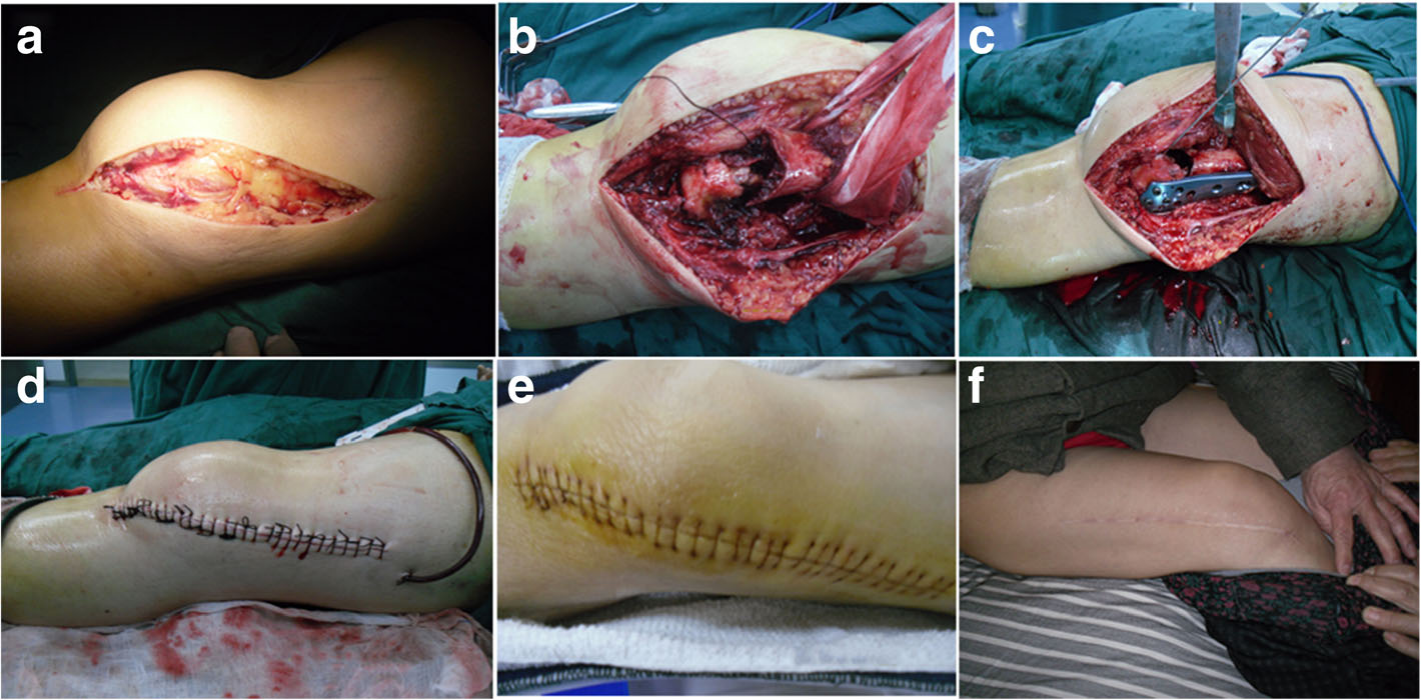
Surgical procedures and postoperative functional exercise. **a** The distal femoral incision was extended to the anterolateral part of the knee joint. **b** The distal femoral fractures was fully revealed and attention should be paid to preserve the integrity of the suprapatellar bursa. **c** Distal femoral compression locking plate was used for solid fixation. **d** The incision was sutured and a drainage tube was placed in the incision. **e** The suture was removed according to the wound healing 12~14 days after surgery. **f** Postoperative functional exercise

After spinal anesthesia, the electric tourniquet was fixed on the suffered limb. A lateral incision of the distal femur extending to the anterior lateral of the knee was obtained. The skin, subcutaneous tissue, deep fascia, and knee joint capsule were cut successively, and the vastus lateralis muscle was revealed (Fig. 2a). The femoral lateral muscle was bluntly separated along its trailing edge. To fully reveal the distal femoral fracture, the periosteum was cut open and stripped. Attention should be paid to protect the integrity of the suprapatellar bursa during the above step (Fig. 2b). For distal femoral fracture type C, the condylar fractures and the anatomical structure of the articular surface of the femoral condyle was restored firstly. For distal femoral fracture type A, temporary fixation was performed by using Kirschner wire, and then hollow screw fixation was conducted after obtaining a satisfactory position. After confirming the fixation, the fracture of the femoral condyle was reset; line of force and the length of the affected limb were restored. Bone graft in stage I was performed for patients with supracondylar comminuted fracture. After the reduction of the fracture, a locking compression plate was used for the fixation of the distal femur (Fig. 2c). C-arm X ray fluoroscopy of distal femur joint surface and internal fixation was conducted to check the line of force, the joint surface, position of steel plate, and screw length. The incision was washed with physiological saline and then sewn up. A drainage tube was placed in the incision, and pressure dressing was performed (Fig. 2d).

### Postoperative management

No external fixation was used after the operation. Routine intravenous antibiotic infusion was performed on the patients for 1 day to prevent infection. The balance of water and electrolytes was monitored carefully postoperatively. Routine blood review was performed on patients with much intraoperative bleeding or postoperative drainage fluid. Symptomatic treatment of blood transfusion was conducted in patients with hemoglobin below 8 g/L. Low-molecular-weight heparin was used to prevent deep venous thrombosis of lower extremities 24 h after the operation. The drainage tube was removed 48 days after the operation, and the incision was removed and sutured according to the condition of incision healing 12~14 days after the operation (Fig. 2e).

### Functional exercise

The initiative activity of limb ankle and toe and isometric contraction of quadriceps femoris was performed 6 h after operation (Fig. 2f). Appropriate knee flexion and extension exercise was performed after limb pain relief to promote the secretion of synovial joint capsule and prevent joint stiffness caused by soft tissue contracture. Passive activity should be delayed for patients with unstable comminuted fractures, such as type C3 distal femoral fractures. Pain reliever should be given for patients with poor exercise compliance and fear of pain. The weight-bearing time was adjusted according to the stability of fracture fixation, the general condition of the patients, and the healing situation of the limbs.

## Results

### Baseline data

Nine males and 11 females with a mean age of 44 ± 3.7 (22~77) years were included in our study. The causes of injury were vehicular accidents (*n* = 8), falls (*n* = 11), and crush injuries (*n* = 1). According to the AO/OTA classification, the fractures were classified as types A_1_ (*n* = 10), A_2_ (*n* = 7), A_3_ (*n* = 10), C_1_ (*n* = 10), C_2_ (*n* = 10), and C_3_ (*n* = 14). General information of the patients are listed in Table 1.

**Table 1:** General information of the patients

Item	
Gender (male/female)	9/11
Mean age (years)	44 ± 3.7
Fracture region (left/right/bilateral)	10/8/2
Injury cause	
Vehicular accidents	8
Fall injuries	11
Crush injuries	1
AO/OTA classification (A_1_/A_2_/A_3_/C_1_/C_2_/C_3_)	2/2/6/1/7/2
Fracture type (open fracture/closed fracture)	3/17
Complications (multiple injuries/chest injury/ brain injury)	2/1/1

### Postoperative outcomes

**Fig. 3:**
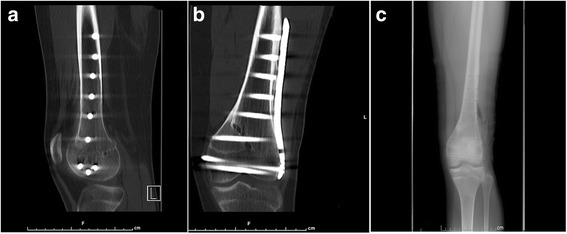
Six-month postoperative images of CT scans and X-rays. **a**, **b** Six-month postoperative CT scans anteroposterior and lateral views showing fracture consolidation. **c** Six months postoperative X-rays anteroposterior views of knee joint showing fracture consolidation after the removal of internal fixation

All patients were followed up for an average of 12 months (6~18 months), and all incisions healed by first intention. Nineteen patients had fracture healing (Fig. 3) 3 to 4 months after surgery; 1 case of distal femoral C_3_ comminuted fracture patient had fracture healing 15 months surgery and received stage II bone grafting and the healing rate of fracture was 95%.

The postoperative joint function was graded according to the Kolmert functional criteria: 14 cases excellent, 4 cases good, 1 case fair, and 1 case poor. All knees reached the state of straightening, and the postoperative excellent rate was 90%. Among them, 8 patients had maximal flexion of more than 120°, 10 patients had flexion between 90° and 120°, and 2 other patients had flexion of 70° and 40°. Fixation of the fracture using a locking compression plate through a posterolateral approach seemed to be an acceptable surgical option for treatment of distal femoral fractures.

## Discussion

High-speed vehicular accidents are responsible for distal femur fractures commonly observed in the young and middle aged. Low energy mechanisms such as fall at home may be responsible for producing fractures of distal femur in the elderly osteoporotic population especially women [2]. Distal femoral fractures are often multi-fragmentary and/or intra-articular and are subjected to muscular forces that render non-operative treatment a poor option. These factors also place high demands on any surgical implant used to fix these fractures and may lead to failure. Pain, decreased range of motion, and compromised function of the knee joint is a common problem arising out of articular incongruity and improper fixation of articular fragments in such fractures [13]. Distal femur locking plate is still the way forward for treating distal femur fractures. Positive results have been published by researchers with implants such as distal femur nail, dynamic condylar screw, and even addition of a medial plate to a distal femur locking plate for treating distal femur fractures.

At present, the surgical treatment of distal femoral fractures is mainly through the lateral approach and anterolateral approach. However, the internal condyle and articular surface of femur cannot be fully exposed in the lateral approach, so it is difficult to reset the articular cartilage surface accurately. At the same time, the unevenness or fixed infirmness of the residual articular cartilage can lead to traumatic arthritis or dysfunction of the knee joint. The lateral approach may also damage the iliotibial band, resulting in lateral instability of the knee after operation [14–16]. Sher et al. [15] reported that the injury of the tibial tunnel in the lateral approach is likely to result in the instability of the knee joint. In the anterolateral approach, the femoral rectus was separated from the vastus lateralis to reveal the vastus intermedius muscle. The vastus intermedius, periosteum, and the joint capsule were cut longitudinally. To reveal the distal femoral fractures, the rectus femoris, vastus intermedius muscle, and patella were pulled to the medial side. However, it dissected the musculi vastus intermedius, which may result in severe postoperative scar healing and thus greatly affect the contraction of the quadriceps femoris [14]. In the posterolateral approach, the vastus lateralis and the biceps femoris were bluntly separated, and the fracture ends were fully exposed by tractioning the vastus lateralis forward. This operation is simple and time-saving, and the integrity of the vastus lateralis is well protected. This technique avoids the destruction of the local blood flow of the fracture ends caused by the incision of the vastus lateralis muscle. At the same time, this technique can avoid the swelling of the postoperative limb caused by the severe destruction of the local soft tissue. The postoperative pain is lighter, which is conducive to early functional exercise. In this study, the posterolateral approach was used to treat the distal femoral fractures, the injury of the vastus intermedius was avoided, and the integrity of the knee extensor device was protected.

While intramedullary nails are well suited to fix extramedullary and simple articular fractures (C1), plates can also be used to treat complex articular fractures. Nevertheless, any displaced articular fracture component must still be anatomically reduced by an open approach and fixed with absolute stability. Fracture fixation was predominantly with anatomical periarticular locking plates, and a smaller number of retrograde intramedullary nails. Whilst this reflects the current literature, with the role of locking plates expanding as the technology evolves [17], studies supporting both methods of fixation have been published [2, 18–23]. Locking compression plates (LCPs), which provide angular stability by minimizing interference with the fracture site, have been used for treatment of distal femoral fractures in osteopenic bone [24, 25]. In recent years, LCPs have been increasingly used for treating metaphyseal comminuted fractures [26]. In contrast to conventional screw-plate systems that depend on the bone plate interface for stability [27], LCPs have been designed with a fixed-angle construct, enabling placement of the plate without any contact with bone [25, 28–30]. With associated insertion guides, these plates can be inserted and fixed by minimally invasive techniques, as in the present cases [26]. These characteristics of LCPs facilitate closed reduction of these fractures and preservation of the blood supply at the fracture site. LCPs have improved fixation strength, pull-out strength of locking screws, and purchase in osteoporotic bone [25, 26, 28, 29]. Because of these advantages, LCPs were helpful in our patients with distal femoral fractures.

This study has several limitations. Firstly, the retrospective design has its inherent limitations of such study. Another limitation in our study is the short follow-up period and lack of control group. Therefore, a hospital-based case–control study to compare the clinical outcome between our techniques and the standard method with long-term follow-up is required in future.

## Conclusions

Fixation of the fracture using a locking compression plate through a posterolateral approach seemed to be an acceptable surgical option for treatment of distal femoral fractures.

## Abbreviations

ABP: Angle blade plate
CBP: Condylar buttress plates
DCS: Dynamic condylar screw
IMNs: Intramedullary nails
